# F200. ELUCIDATING THE ROLE OF CILIA IN NEUROPSYCHIATRIC DISEASES THROUGH INTERACTOME ANALYSIS

**DOI:** 10.1093/schbul/sby017.731

**Published:** 2018-04-01

**Authors:** Madhavi Ganapathiraju, Srilakshmi Chaparala, Cecilia Lo

**Affiliations:** University of Pittsburgh

## Abstract

**Background:**

Cilia are microtubule-based organelles present on the surface of many eukaryotic cell types critical for tissue homeostasis and proper organ development. Ciliary dysfunction underlies a growing list of human diseases and disorders collectively called ciliopathies such as Bardet-Biedl syndrome (BBS), Joubert syndrome, Meckel–Gruber syndrome and primary ciliary dyskinesia. Many ciliary proteins are associated with neuronal function consistent with neuronal developmental delays, cognitive, learning, and memory deficits observed in several ciliopathies, suggesting that ciliary dysfunction may contribute to pathogenesis of neuronal diseases and that an understanding of how ciliary proteins function together as a system would provide much needed mechanistic insights into their molecular etiologies.

**Methods:**

We constructed protein-protein interaction (PPI) networks of genes associated with cilia and those associated with 7 neuropsychiatric diseases: schizophrenia, attention deficit hyperactivity disorder, major depressive disorder, bipolar disorder, autism spectrum disorder, Alzheimer’s disease and Parkinson’s disease. The interactome is constructed with experimentally determined PPIs from BioGRID and HPRD databases and novel PPIs predicted using our High-confidence PPI Prediction (HiPPIP) model. We previously presented Schizophrenia Interactome constructed using HiPPIP andalso showed that novel PPIs are highly accurate based on computational and experimental validations. We validated additional PPIs of cilia interactome here. We computed how closely connected cilia is to genes associated with neuropsychiatric diseases, through interactome and pathway analysis. Additionally, we analyzed drugs that proteins in the cilia interactome, and found that majority of these drugs are nervous system associated drugs.

**Results:**

The ciliary protein interactome consists of 165 ciliary proteins with 1,011 known PPIs and 765 novel PPIs. We found the overlap between cilia and neuropsychiatric interactomes to be statistically highly significant. For e.g., cilia interactome has an overlap of 125 genes with schizophrenia interactome of which 26 are novel interactors of cilia, and has significant overlap with pathways relevant to schizophrenia. About 184 genes in the cilia interactome are targeted by 548 FDA approved drugs, of which 103 are used to treat nervous system diseases.

**Discussion:**

Ciliary genes like DRD1 and DRD2 are implicated in neurotransmission and associated with schizophrenia. DRD1 has 4 novel interactors and DRD2 has 12 novel interactors that may have significant role in the pathology of mental disorders. Neuronal pathways associated with cilia interactome with high statistical significance such as dopamine signaling, eNOS signaling, synaptic long-term potentiation pathways are known to be associated schizophrenia. Wnt signaling and PCP signaling are also known to be associated with cilia mediated neurodevelopmental signaling, defects in these pathways contributing to schizophrenia. Novel interactions for cilia proteins validated by experiments have functional significance in association with cilia and neuronal disorders. For e.g., IFT88, a cilia protein required for cilia assembly, is critical for SHH signaling, cell cycle regulation and cerebellar development and is also associated with schizophrenia and bipolar disorder. CACNA1I is predicted to interact with DNAL4 and MKS1, both involved in transport of proteins required for ciliogenesis. GWAS studies show that CACNA1I is associated with schizophrenia. Taken together, the cilia interactome presented here provides novel insights into the relationship between ciliary protein function and neuropsychiatric diseases.

